# Surface-enhanced Raman scattering by colloidal CdSe nanocrystal submonolayers fabricated by the Langmuir–Blodgett technique

**DOI:** 10.3762/bjnano.6.245

**Published:** 2015-12-14

**Authors:** Alexander G Milekhin, Larisa L Sveshnikova, Tatyana A Duda, Ekaterina E Rodyakina, Volodymyr M Dzhagan, Ovidiu D Gordan, Sergey L Veber, Cameliu Himcinschi, Alexander V Latyshev, Dietrich R T Zahn

**Affiliations:** 1 A.V. Rzhanov Institute of Semiconductor Physics, pr. Lavrentieva 13, Novosibirsk 630090, Russia; 2 Novosibirsk State University, Pirogov str. 2, Novosibirsk 630090, Russia; 3 Semiconductor Physics, Technische Universität Chemnitz, 09107 Chemnitz, Germany; 4 International Tomography Center SB RAS, Novosibirsk 630090, Russia,; 5 Institut für Theoretische Physik, TU Bergakademie Freiberg, 09596 Freiberg, Germany

**Keywords:** CdSe nanocrystals, dimers, localized surface plasmon resonance, metal nanoclusters, phonons, surface-enhanced Raman spectroscopy

## Abstract

We present the results of an investigation of surface-enhanced Raman scattering (SERS) by optical phonons in colloidal CdSe nanocrystals (NCs) homogeneously deposited on both arrays of Au nanoclusters and Au dimers using the Langmuir–Blodgett technique. The coverage of the deposited NCs was less than one monolayer, as determined by transmission and scanning electron microscopy. SERS by optical phonons in CdSe nanocrystals showed a significant enhancement that depends resonantly on the Au nanocluster and dimer size, and thus on the localized surface plasmon resonance (LSPR) energy. The deposition of CdSe nanocrystals on the Au dimer nanocluster arrays enabled us to study the polarization dependence of SERS. The maximal SERS signal was observed for light polarization parallel to the dimer axis. The polarization ratio of the SERS signal parallel and perpendicular to the dimer axis was 20. The SERS signal intensity was also investigated as a function of the distance between nanoclusters in a dimer. Here the maximal SERS enhancement was observed for the minimal distance studied (about 10 nm), confirming the formation of SERS “hot spots”.

## Introduction

Since its observation in 1974 [1], surface-enhanced Raman scattering (SERS) has become a powerful technique for detecting and studying ultra-low quantities of organic and biological substances [2–7] down to a single molecule [8–9]. The primary benefit of SERS is that the intensity of Raman scattering by vibrational modes in molecules is drastically increased (typically by a factor of 10^5^–10^6^) when the molecules are placed in the proximity of noble metal nanoclusters or on rough metal surfaces. The locally enhanced electromagnetic field induced by the localized surface plasmon resonance (LSPR) in the vicinity of metal surface is responsible for the Raman scattering intensity enhancement, which is proportional to the fourth power of the enhancement of the local ﬁeld [3–7]. The progress in controlled nanostructuring of metal surfaces has led to the development of high-performance SERS substrates with an average SERS enhancement factor (EF) well above 10^6^ (EF > 10^8^) for ultrasensitive analysis of organic substances [10–12]. It was also shown that for single molecular detection, the EF can reach an ultimate value of 10^14^–10^15^ [8–9].

However, with few exceptions (such as carbon-based materials [13–16]), inorganic nanostructures have been much less investigated by SERS. It was already shown that several types of semiconductor NCs, including CdS [17–18], CdTe [19], CdSe [20–24], ZnO [25–28], GaN [26], and Cu*_x_*S [29–30], reveal the SERS effect by optical phonons when placed in close proximity of Au or Ag nanoclusters.

Among those, CdSe NCs have attracted much attention for SERS experiments for at least two reasons. From one side, colloidal CdSe NCs are already used in commercial applications [31]. The information on the crystal structure of the NCs, their size, shape, and mechanical strain (which can be derived from the frequencies of the Raman phonon modes as seen in SERS spectra) is crucial for device performance. From other side, CdSe NCs are resistant against intense laser irradiation and have a direct band transition energy located in the same (red) spectral range as that for LSPR in Au nanoclusters and are therefore considered as a model system for resonant SERS experiments.

SERS by optical phonons was previously observed for several CdSe-based NCs, including pure CdSe and core–shell CdSe/CdZnS NCs deposited on Au or Ag substrates of various morphology [20–23]. Resonant SERS enables the observation of LO phonon modes of the CdSe core in a monolayer of core–shell CdSe/ZnS NCs deposited on commercially available SERS substrates [20]. However, the usage of conventional SERS conditions provides a SERS enhancement that is insufficient for investigation of an individual nanostructure.

Very recently, it was demonstrated that the phonon spectrum of individual CdSe nanoplatelets can be probed by SERS when the semiconductor nanostructure is placed in the gap between a gold nanocluster and a gold surface (the so-called “hot spot”) [32]. As in the case of metal nanoclusters in close proximity, the plasmonic gap supports electromagnetic fields confined in the gap much that are stronger (typically a few orders of magnitude depending on the gap size) than the field located near a single metal nanocluster or a metal surface [33]. SERS enhancement benefits from the implementation of this experimental geometry. In particular, it allows the influence of the spatial confinement and the structure anisotropy on optical phonon modes in individual CdSe nanoplatelets to be investigated [33].

For the investigation of resonant SERS, it is vitally important to have metal nanocluster arrays with controlled and intentionally varied structural parameters as well as homogeneous NC coverage. While metal nanocluster arrays can be fabricated by means of electron-beam lithography [34–35], nanoimprint lithography [36–37], or nanosphere lithography [38–39], the deposition of homogeneous films of CdSe NCs is possible by using Langmuir–Blodgett (LB) technology [40–43].

In this paper we report on the study of resonant SERS by CdSe NC coverage of less than one monolayer deposited onto regular arrays of Au nanoclusters, with a particular focus on Au dimer arrays.

## Experimental

**Figure 1:**
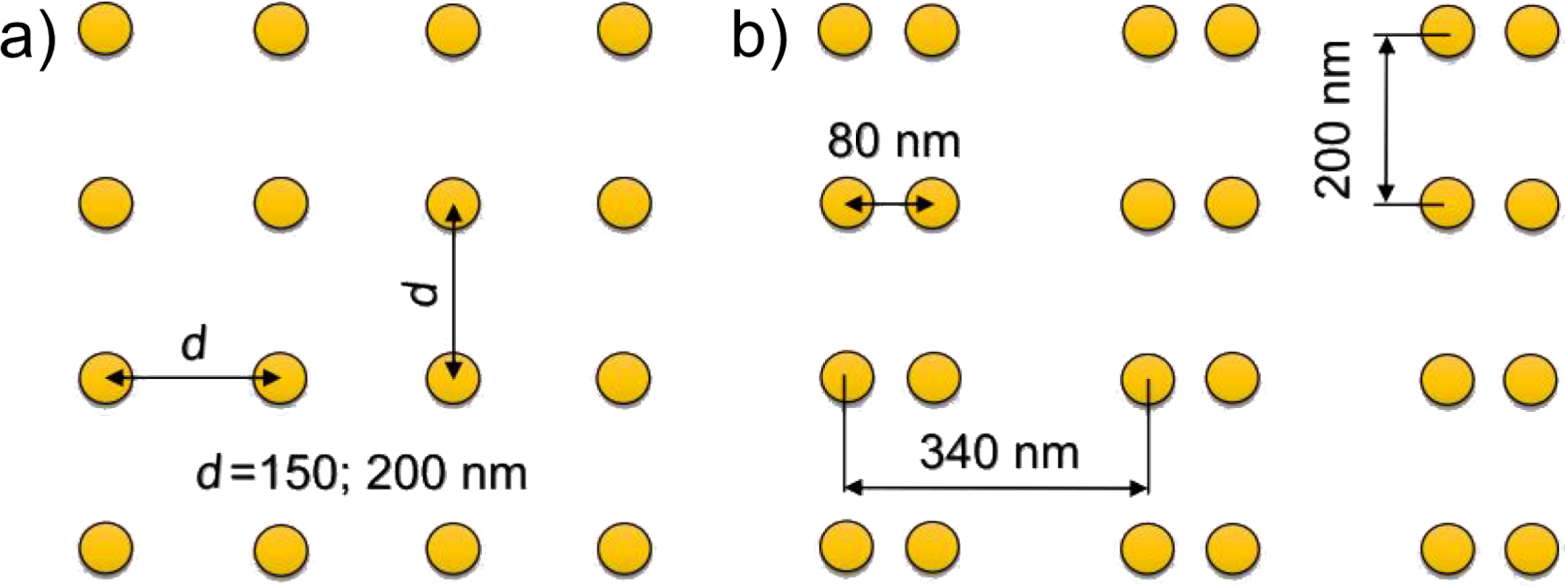
A sketch representing (a) a regular Au nanocluster array and (b) a Au dimer array.

Colloidal CdSe NCs with a diameter of 5.2 nm purchased from Lumidot were homogeneously deposited on specially prepared plasmonic substrates by means of the LB technique, which is traditionally used for the fabrication of both highly ordered organic films [44] and NCs with controlled areal density [18] on a solid substrate [24,29]. The periodic Au nanocluster arrays were fabricated by direct electron beam writing (Raith-150, Germany) on (001)-oriented Si substrates and served as SERS-active substrates. They were fabricated with two areas of Au nanoclusters with pitch of 150 and 200 nm (Figure 1a). Each area contained 30 square, 10 × 10 µm^2^ lattices with different diameters of Au nanoclusters for each lattice, as described in [24,29]. The fabrication details of regular arrays of Au nanoclusters and dimers on a Si substrate are presented in [29]. In addition to regular arrays of Au nanoclusters, arrays of paired Au nanoclusters or dimers were fabricated by electron beam lithography on a Si substrate covered with 75 nm of SiO_2_. The silicon dioxide layer was implemented to combine the benefits of interference-enhanced Raman scattering and SERS for further enhancement of the Raman signal [30]. It is worth mentioning that for regular nanocluster arrays, a silica spacer causes an undesirable shift of LSPR energy from the resonant conditions due to the change of dielectric function of the surrounding media. However, as will be shown later for dimers that have LSPR energy distinct from individual nanoclusters, the resonant conditions are again fulfilled. The distance between the centers of nanoclusters in a dimer was fixed at 80 nm, while the pitch of dimers in the orthogonal directions was 200 and 340 nm, as shown in Figure 1b. The nanocluster size for a dimer was gradually varied from 71 to 40 nm from array to array. Thus, the gap size between the adjacent nanoclusters in a dimer was in the range between 9 and 40 nm. The accuracy in the determination of nanocluster size and gap size between the adjacent nanoclusters in a dimer in the experiments was about ±5 nm, limited by the statistical fluctuation and size of the gold grains (about 10 nm).

The size, shape, and areal density of CdSe NCs were determined by SEM using the same Raith-150 system at 10 kV acceleration voltage, 30 µm aperture, and 6 mm working distance. The high-resolution transmission electron microscopy (HR-TEM) experiments were performed using a JEM-400EX (JEOL) electron microscope with an accelerating voltage of 400 keV. The point resolution was 0.165 nm.

The LSPR energy in Au dimer arrays was determined from reflection measurements carried out using a Bruker Vertex 80v Fourier transform infrared spectrometer supplied with a Hyperion 2000 infrared microscope in the spectral range from 400–1000 nm with an aperture of 10 µm. The reflection from a part of the same substrate but without dimer arrays was used as a reference.

Micro-Raman experiments were performed with a LabRam spectrometer in backscattering geometry at 300 K. The excitation wavelength of λ_exc_ = 632.8 nm (provided by a He–Ne laser) was used in the Raman experiments. The laser light incident on the sample surface was focused to a ≈1 µm spot diameter with a power of about 0.5 mW. Raman experiments of CdSe NCs deposited on dimer arrays were carried out with the incident and scattered light polarized parallel or perpendicular to the long axis of the dimers.

## Results and Discussion

### CdSe NCs on regular arrays of Au nanoclusters

**Figure 2:**
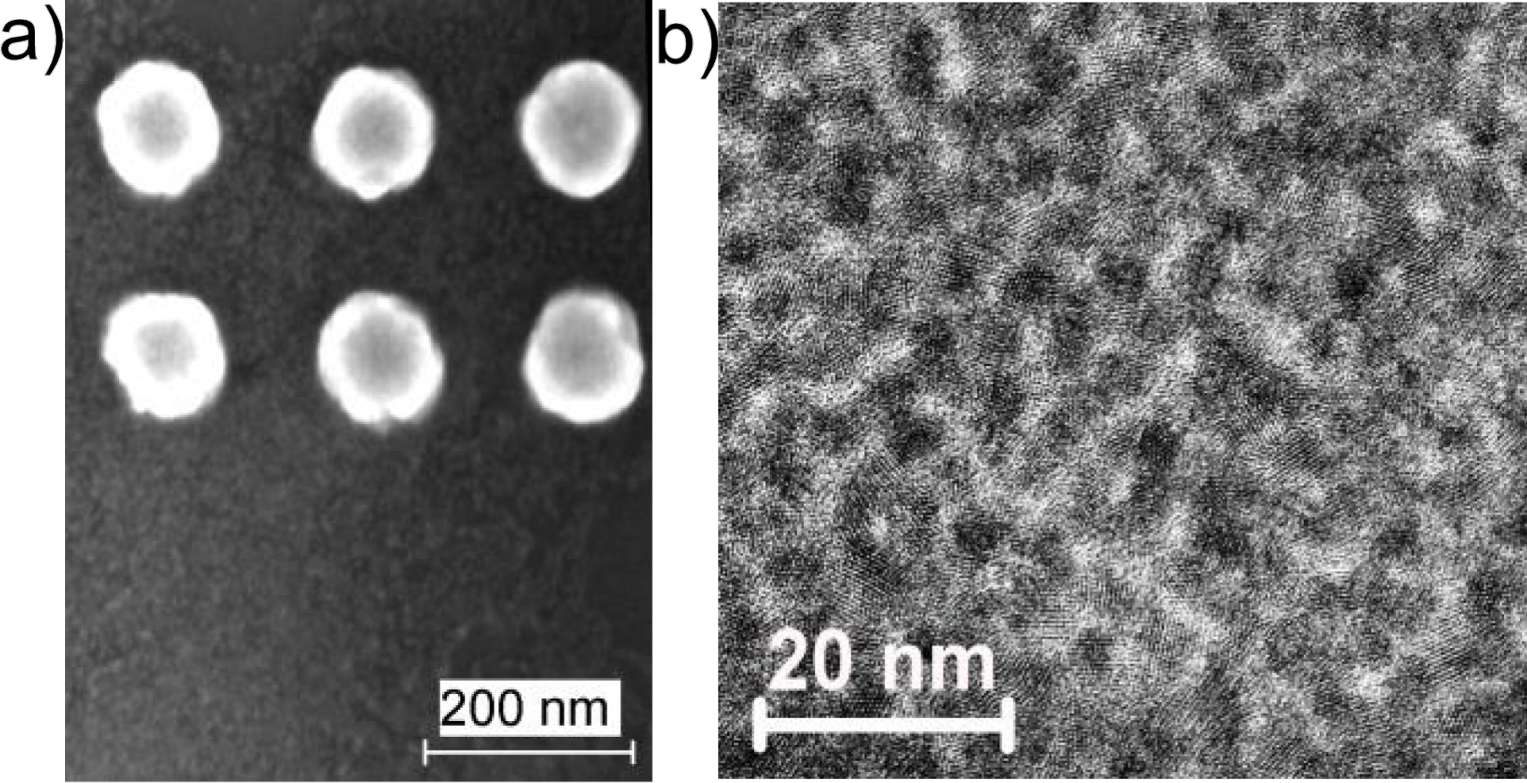
(a) SEM image of a single monolayer of CdSe NCs deposited on Si (bottom) and Au nanocluster arrays with a pitch of 200 nm (top). (b) HR-TEM image of a single monolayer of CdSe NCs formed on a carbon-coated Cu grid.

Typical SEM and HR-TEM images of a single monolayer of CdSe NCs deposited by the LB technique on the plasmonic substrate and on a carbon-coated Cu grid are shown in Figure 2. This demonstrates a dense, homogeneous coverage of the NCs for both the Si substrate with a Au nanocluster array and the Cu grid. The Raman spectrum acquired from the area where CdSe NCs are deposited on the Si substrate reveals only features inherent to crystalline Si.

**Figure 3:**
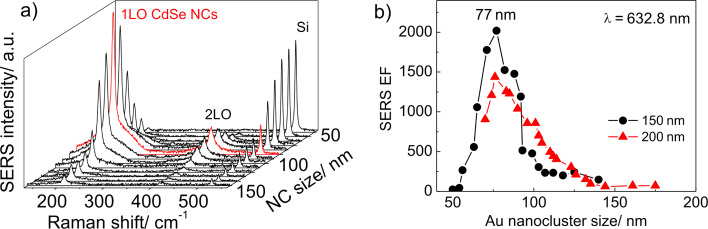
(a) SERS spectra of a single monolayer of CdSe NCs deposited on the nanocluster arrays with decreasing Au nanocluster size. (b) The SERS EF of LO phonon modes in CdSe NCs as a function of Au nanocluster size for Au nanocluster arrays with a pitch of 150 and 200 nm.

However, the Raman spectra of CdSe NCs deposited on the nanocluster arrays (Figure 3a) reveal a pronounced peak at about 207.5 cm^−1^ (denoted as 1LO in Figure 3a) and weaker peaks at multiple frequencies (about 415 and 623 cm^−1^), which are attributed to a confined longitudinal optical (LO) mode and its overtones from CdSe NCs [45]. From this point on, the variation of 1LO phonon mode in CdSe NCs deposited on various substrates does not exceed 0.6 cm^−1^. The appearance of new Raman peaks illustrates the SERS effect by optical phonons in CdSe NCs. The mode at 520.5 cm^−1^ originates from the optical phonons of the Si substrate. With decreasing Au nanocluster size, the SERS intensity of CdSe-related modes shows a maximum resonant behavior for a Au nanocluster size of about 77 nm, while the intensity of the Si phonon mode gradually increases. The behavior of the 1LO mode of the CdSe NCs deposited on the Au nanocluster arrays of different nanocluster pitches (*d* = 150 and 200 nm) is rather similar (Figure 3b) and confirms the resonant character of SERS by optical phonons in CdSe NCs. As was shown from the micro-ellipsometry measurements [24], the LSPR energy in the array of 77 nm Au nanoclusters was about 625 nm. This value is very close to the excitation energy used in the SERS experiment (632.8 nm) that leads to the resonant SERS. Note that in the case of CdSe NCs on Au nanocluster arrays, the double resonance condition is fulfilled when the excitation energy matches both the transition energy in the NCs (2.03 eV or 610 nm) and the LSPR energy in the Au nanoclusters.

The increase of the Si phonon mode intensity with decreasing Au nanocluster size is due to a larger proportion of bare (not covered with Au nanoclusters) Si surface for the arrays with Au nanoclusters of smaller size.

A broad band between 170 and 260 cm^−1^ which appears pronounced in the SERS spectra (Figure 3a) measured in the resonant conditions will be further discussed in detail. From this point, a constant background was subtracted from the SERS spectra.

The resonance behavior of SERS by optical phonons for different excitation wavelengths in regular Au arrays with a period of 150 nm was investigated in detail in our previous work [24]. It was previously shown [24] that the SERS EF (determined for Au nanocluster arrays with a pitch of 150 nm) as a function of nanocluster size has a maximum of about 2 × 10^3^. By increasing the nanocluster pitch up to 200 nm, the SERS intensity decreases due to the decreased number of Au nanoclusters. Note that for both Au array periods, the maximal EF is observed for a Au nanocluster size of 77 nm indicating the noninteracting character of neighboring Au nanoclusters [24]. The Raman intensity of the 1LO phonon mode in the spectra of CdSe NCs deposited with the same process on a Si substrate without Au nanoclusters was below the noise level. A detectable Raman phonon intensity was obtained when the acquisition time was increased up to 60 s and was further used as a reference.

### CdSe NCs on Au dimer arrays

**Figure 4:**
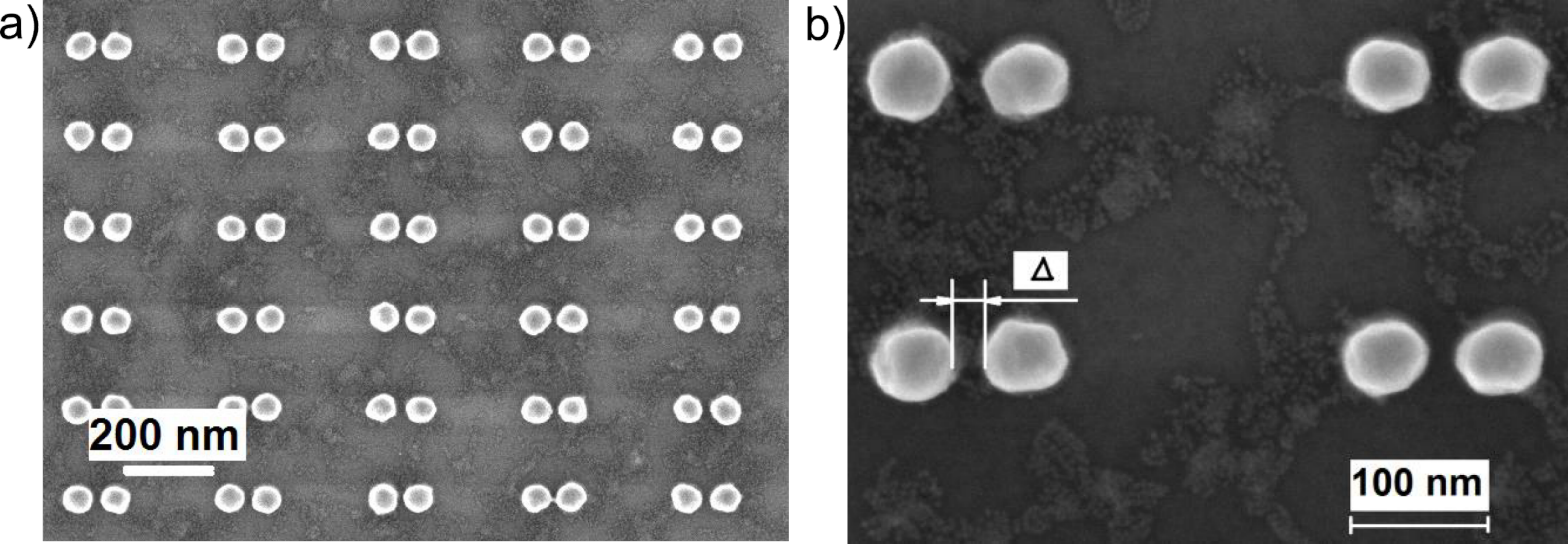
(a) SEM image of submonolayer coverage of CdSe NCs deposited on a Au dimer array. (b) An enlarged fragment of the SEM image of the structure shown in (a). The dimer gap is denoted as Δ.

To achieve further SERS enhancement, CdSe NCs were deposited on arrays of Au dimers (Figure 4a). The SERS experiments with Au dimer arrays allow for the reduction of the areal density of the CdSe NC coverage (as shown in Figure 4b) without reduction of the SERS signal. This is due to the formation of localized electric field hot spots within the dimer gap, which is partially filled with CdSe NCs.

In comparison with Au nanocluster arrays, a dimer array represents an anisotropic plasmonic structure which is characterized by two coupled resonance plasmon modes when excited perpendicular to the structure surface [46]. The longitudinal LSPR mode (which is polarized along the long dimer particle axis) red-shifts with decreasing gap between the nanoclusters in a dimer. The other, transverse plasmon mode (polarized in the orthogonal polarization) blue-shifts very slightly with decreasing gap.

**Figure 5:**
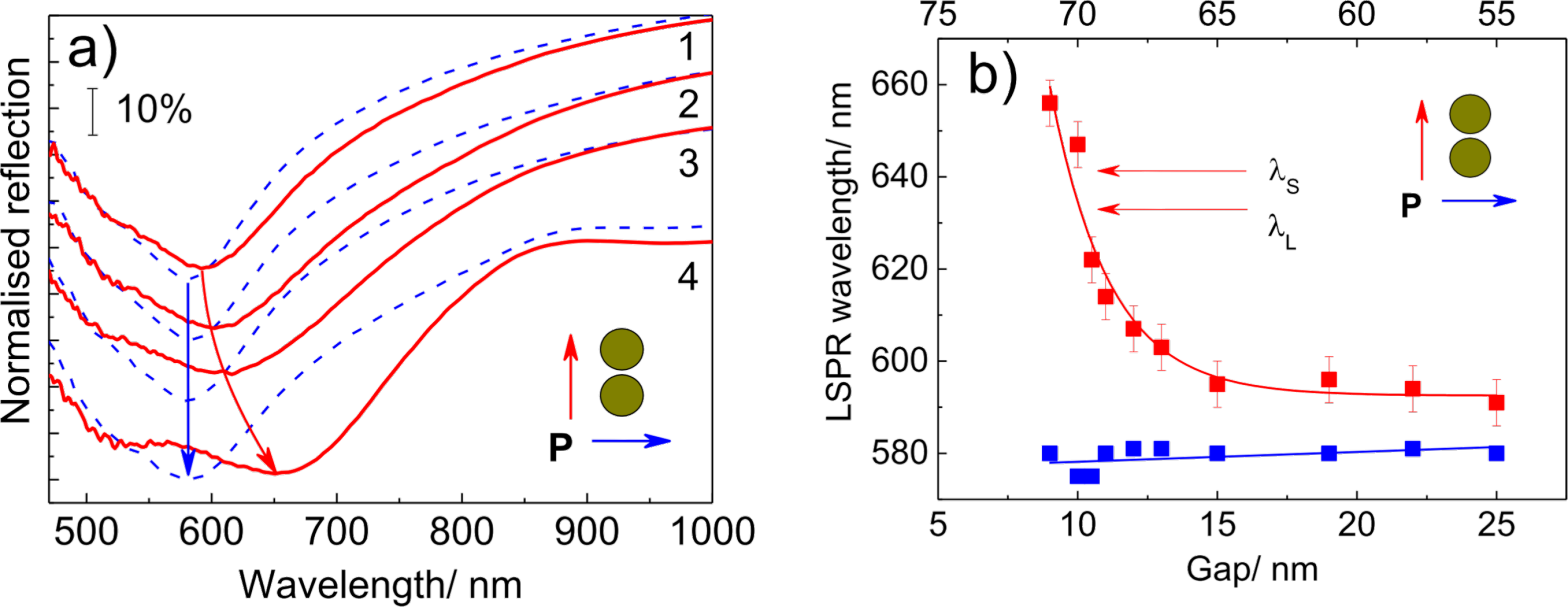
(a) The representative reflection spectra of Au dimer arrays with decreasing gap size (Δ = 15 cm, 1; Δ = 13 cm, 2; Δ = 10 cm, 3; Δ = 8 cm, 4) measured with light polarized parallel to (solid) and perpendicular to (dashed) the long dimer axis. (b) The dependence of the LSPR wavelength on the gap size, Δ, between dimers. The arrows show the laser excitation wavelength, λ_L_, and the wavelength of the scattered photons, λ_S_.

The LSPR energy in the Au dimer arrays was determined by micro-reflection measurements using linearly polarized light. The reflection spectra taken from Au dimer arrays with the same distance between the nanocluster centers (80 nm) (but different nanocluster sizes and, thus, different gaps between dimers) are presented in Figure 5a. The reflection spectra taken on areas without Au nanocluster arrays measured with the corresponding polarizations were used as reference spectra. One can see from the figure that the reflection spectra measured with polarization parallel to the long axis of the dimers reveal a pronounced minimum at about 590 nm for Δ = 15 nm. This corresponds to a red-shift with decreasing gap size, reaching a value of about 655 nm for Δ = 8 nm. However, the minimum at 580 nm measured with the polarization perpendicular to the long dimer axis for dimers with a nanocluster size of 65 nm and a gap size Δ = 15 nm barely changes when the gap size is varied. These minima are attributed to the coupled LSPR modes in dimers as described above. The variation of the LSPR wavelength with gap size agrees well with the universal scaling behavior previously reported [46]. The shoulder seen in the reflection spectra at about 520 nm (2.38 eV) for both polarizations is due to the interband transitions in gold [47].

The data on the LSPR wavelength derived from the reflection measurements are summarized in Figure 5b. As can be seen, the LSPR wavelength of the dimers with the smallest gap size (about 10 nm) is very close to the excitation wavelength (λ_L_ = 632.8 nm). Relative to that of the LO phonon mode frequency of 207.5 cm^−1^ (about 26 meV), the wavelength of the scattered photons is somewhat higher (λ_S_ = 641.4 nm) and even closer to the LSPR wavelength (Figure 5b). Consequently, between these two values, λ_L_ and λ_s_, the conditions for the ultimate resonant SERS are fulfilled for which the maximal SERS enhancement factor is expected [48].

**Figure 6:**
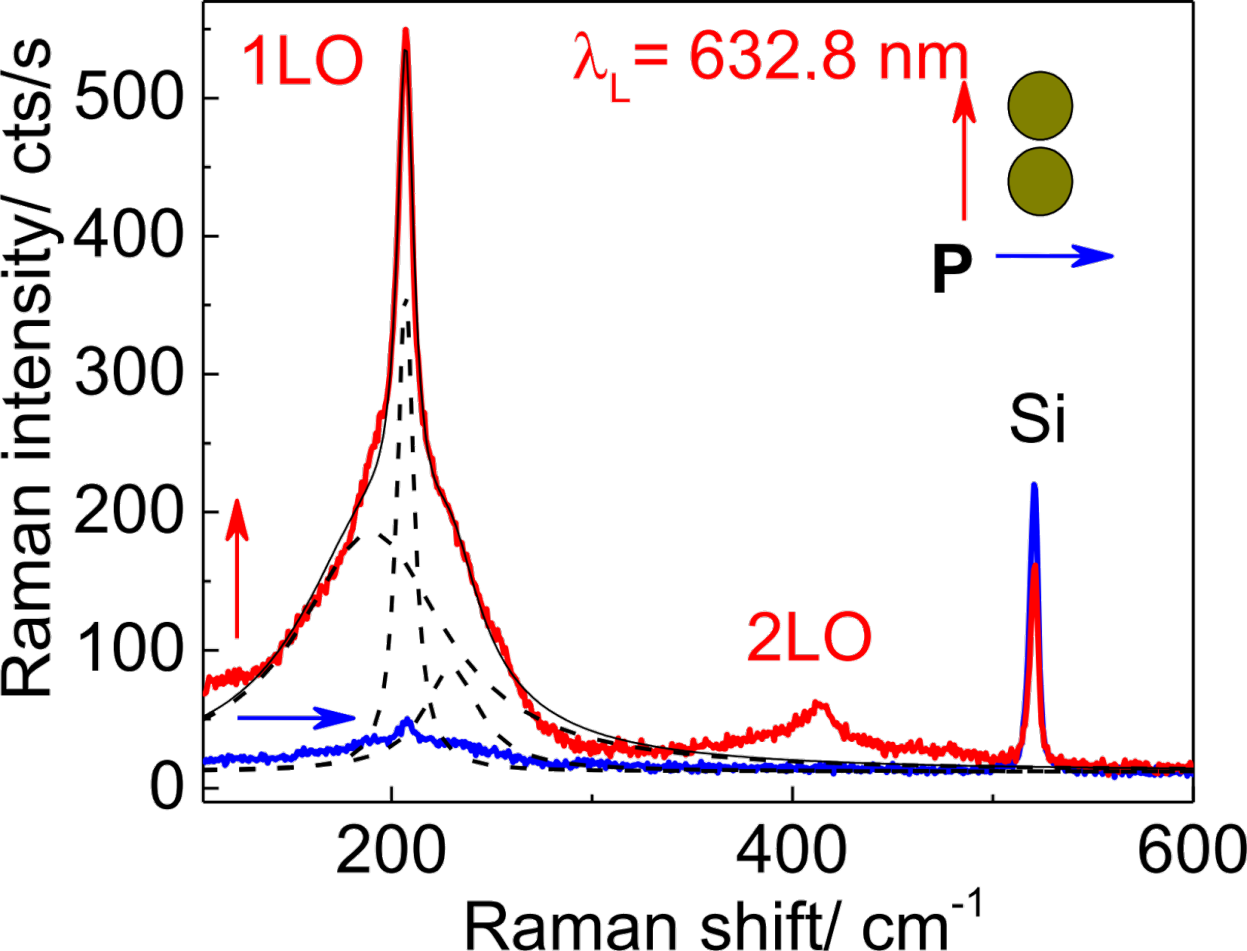
SERS spectra of a submonolayer of CdSe NCs deposited on the dimer array with a gap size of about 10 nm measured at λ_L_ = 632.8 nm with parallel (vertical arrow) and perpendicular (horizontal arrow) polarized light with respect to the long dimer axis. The Lorentzian curves used for the fit of the Raman spectra as well as the fitting result are shown by dashed and solid lines, respectively.

Indeed, the SERS spectra of CdSe NCs on Au dimers with the smallest gap between nanoclusters measured with 632.8 nm excitation wavelength and light polarized parallel to the long dimer axis reveal the most intense LO phonon mode. This mode appears at 207.5 cm^−1^ (Figure 6) superimposed with a broad background between 170 and 260 cm^−1^ and a weaker feature due to 2LO scattering near 414 cm^−1^. The intensity of the LO mode decreases by a factor of 20 in the orthogonal geometry (calculated after background subtraction). The background was fit by two Lorentzian curves centered at about 190 and 230 cm^−1^. The first can be attributed to scattering by surface optical modes, which was previously well-investigated in CdSe NCs [45,49–50]. The latter may originate from SERS by amorphous selenium formed on the NC surface due to partial photodegradation of CdSe NCs under laser illumination [51–52]. This mode has somewhat different intensity and shape for different Au arrays and depends on the sample preparation history. The Lorentzian curves obtained as a result of the fitting procedure are shown in Figure 6.

Interestingly, the intensity of the Si phonon mode seen in the spectra polarized parallel to the dimer axis is 30% weaker than that for the orthogonal polarization. This effect could be explained by the resonant absorption of the laser light by the longitudinal plasmon near Au dimers that reduces the Raman scattering signal from the underlying Si. The observed anisotropy of both CdSe- and Si-related phonon modes gradually decreases with increasing the gap size and disappears for gap sizes above 20 nm. This means that dimer arrays with relatively large gap sizes behave very similar to the regular Au nanocluster arrays.

**Figure 7:**
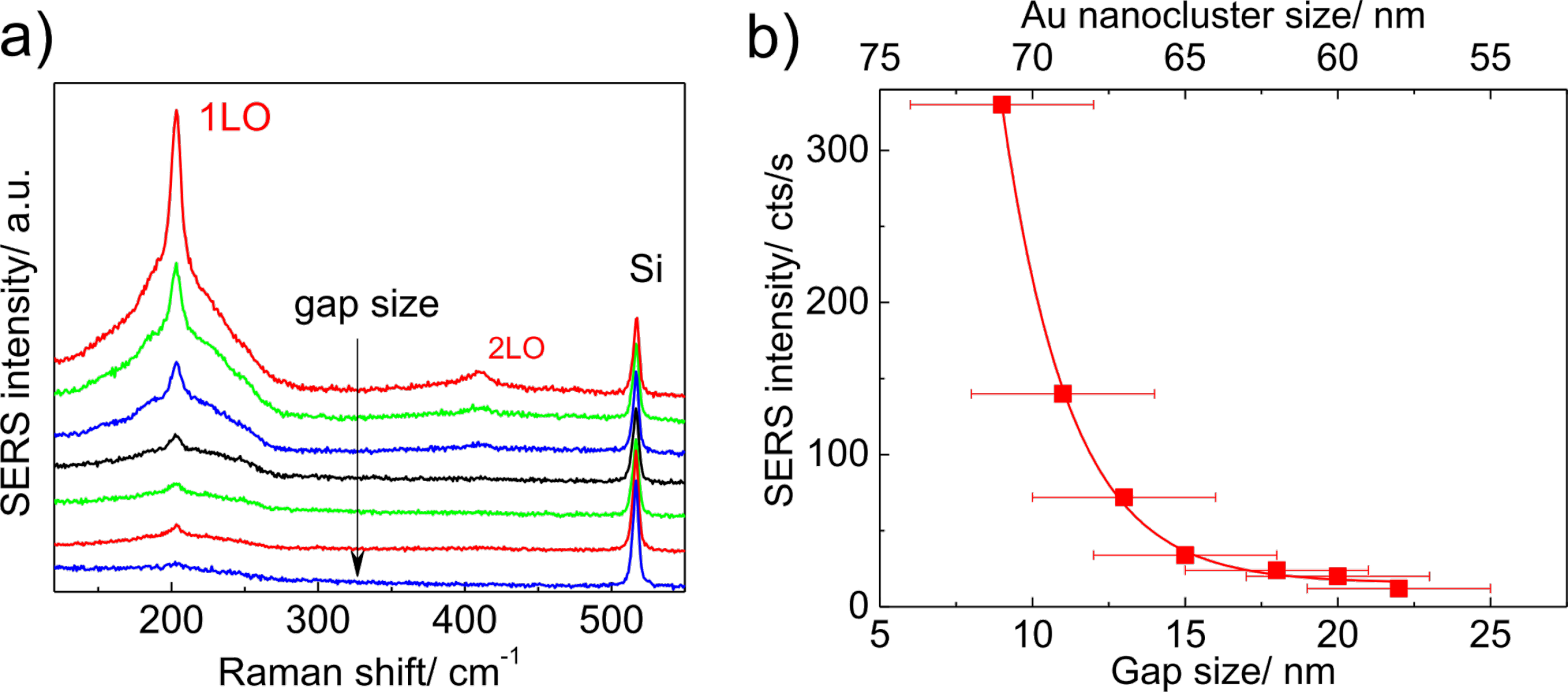
(a) SERS spectra of a submonolayer of CdSe NCs deposited on dimer arrays with increasing gap size measured at λ_L_ = 632.8 nm with light polarized parallel to the long dimer axis. (b) The dependence of SERS intensity on the gap size and Au nanocluster size.

The intensity of the LO mode for the excitation polarized parallel to the long dimer axis as well as its overtone, 2LO, decreases with increasing gap size between Au nanoclusters (Figure 7a,b). The decrease of the CdSe phonon modes is accompanied by an increase of Si peak by 43% for Au nanocluster size of 55 nm. The decreasing intensity of the LO mode in CdSe is associated with a reduction of the electromagnetic field in the gap between Au nanoclusters responsible for the SERS effect and with detuning the SERS resonance. The relatively large increase of the Si peak cannot be explained only by the decreasing size of the Au nanoclusters (from 70 to 55 nm) and thus larger illuminated area of the bare Si substrate taking part in the Raman process. The Raman signal from Si should be proportional to this area. Simple geometrical consideration gives an increase of the illuminated area with decreasing Au nanoclusters of about 12%, which is sufficiently smaller than the experimentally observed value of intensity increase (43%). This effect can again be explained by the resonant absorption of the laser light by the longitudinal plasmon near the Au dimers with the smallest gap. This causes an increase of the “effective” Au nanocluster size (or extinction cross-section) of up to about 100 nm, and thus the reduction of the Raman scattering signal from the underlying Si. This conclusion is consistent with previously reported results [53] confirming that for noble metal nanoclusters the extinction cross-section can be up to 10 times larger than their geometrical cross-section. The absorption by the plasmon is reduced with decreasing Au nanocluster size, as can be seen in the experiment.

## Conclusion

LB technology was successfully applied for the formation of a homogeneous, submonolayer coverage of CdSe NCs on ordered plasmonic structures fabricated by direct electron beam writing.

The pronounced enhancement of the Raman scattering intensity by optical phonons in the CdSe NC ensembles deposited on regular arrays of Au nanoclusters and Au dimers, which resonantly depends on Au nanocluster size and laser excitation wavelength, provides evidence of the resonant character of the surface-enhanced Raman scattering effect. A maximal SERS enhancement by optical phonons in CdSe NCs was achieved for arrays of Au dimers with a minimal gap between nanoclusters in a dimer resonantly excited with the light polarized parallel to the long dimer axis where hot spots are realized.
